# **Identification of maternal allele sequences of IG-DMR that are** essential for neonatal **viability**

**DOI:** 10.1371/journal.pone.0324882

**Published:** 2025-05-22

**Authors:** Satoshi Hara, Akari Muramatsu, Miho Terao, Shuji Takada

**Affiliations:** 1 Department of Systems Developmental Biology, National Research Institute for Child Health and Development, Tokyo, Japan; 2 Devision of Molecular Genetics & Epigenetics, Department of Biomolecular Science, Faculty of Medicine, Saga University, Saga, Japan; 3 Department of NCCHD, Graduate School of Medical and Dental Sciences, Institute of Science Tokyo, Tokyo, Japan; Fujita Health University: Fujita Ika Daigaku, JAPAN

## Abstract

The expression of imprinted genes in the *Dlk1*-*Dio3* domain is regulated by *Dlk1*-*Meg3* intergenic DMR (IG-DMR), which is methylated in a parental-of-origin-specific manner. An unmethylated 4.1-kb region in the IG-DMR is essential for the maternal allele. Several molecular mechanisms have been proposed for the 4.1-kb region of IG-DMR; however, the sequence in the 4.1-kb region essential for imprinted gene expression is still unknown. To explore the sequence responsible for the IG-DMR *in vivo*, we generated mutant mice with a series of IG-DMR deletions. We observed that a deletion of the 2.7-kb region, including the IG-DMR transcriptional regulatory element (IG^TRE^), on the maternal allele causes IG-DMR dysfunction, resulting in perinatal lethality. At least two functional sequences exist in IG^TRE^ that are functionally redundant *in vivo*, and the paternal transmission of a mutant allele, in which IG^TRE^ was deleted together with a tandem repeat sequence in IG-DMR (IG^Rep^), rescued embryonic lethality due to a lack of paternal IG^Rep^. Our findings revealed that a sequence responsible for the lethal phenotype of the maternally inherited 4.1-kb deletion of IG-DMR is in the IG^TRE^ domain.

## Introduction

In mammals, most genes are transcribed from both paternal and maternal alleles when expressed; however, certain genes, designated as imprinted genes, are expressed mono-allelically based on the parent-of-origin. Most imprinted genes form clusters, and their expression is regulated by an imprinting control region (ICR). Epigenetic regulatory mechanisms, such as DNA methylation, play important roles in the parent-of-origin-specific expression of imprinted genes, and the ICR is in a differentially methylated region in which the DNA methylation state differs in a parent-of-origin-specific manner [1]. Genome-wide DNA methylation, including that in ICRs, establishes sperm- or oocyte-specific patterns in germ cells. Most DNA is actively or passively demethylated during embryogenesis after fertilization, whereas parent-of-origin-specific methylation of ICRs is maintained throughout embryogenesis. DMRs with germline-derived differential methylation are called germline DMRs, while DMRs that acquire parental allele-specific differential methylation post-implantation are called somatic DMRs [2]. Evidence exists for abnormalities in the DNA methylation of DMRs that cause biallelic expression or suppression of imprinted genes, which causes abnormal embryonic development or postnatal defects. Because dysregulation of imprinted gene expression causes congenital diseases in humans (e.g., imprinting disorders), epigenetic regulation of imprinted regions is important for normal development [3].

**Fig 1:**
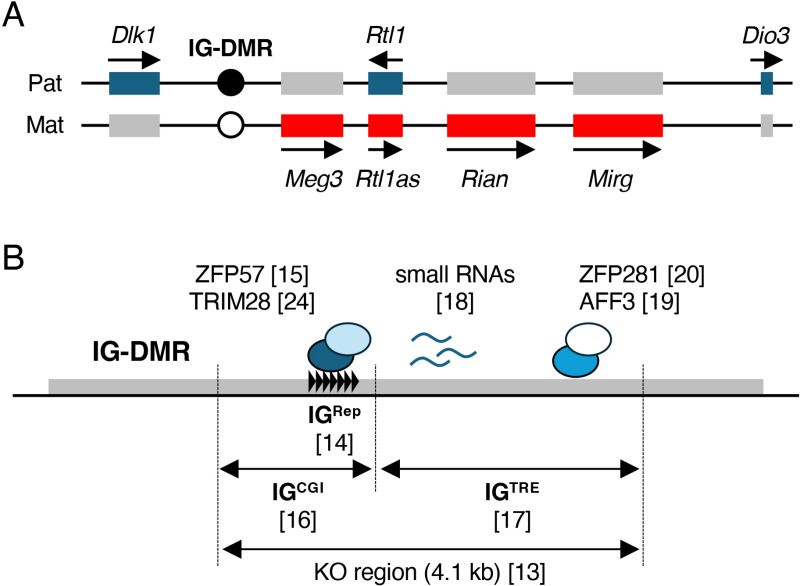
Mouse *Dlk1*-*Dio3* locus and regulatory mechanisms of IG-DMR. (A) Schematic representation of the mouse *Dlk1*-*Dio3* domain. Genomic DNA is indicated by a black line. Imprinted genes expressed by paternal and maternal alleles are shown in blue and red boxes, respectively. Arrows indicate the direction of transcription and expressed alleles. Methylated and unmethylated IG-DMR are shown in black and white circles, respectively. (B) Schematic representation of IG-DMR. The gray box indicates IG-DMR. The repeated unit of IG^Rep^ is indicated by black triangles. Transcriptional factors that bind to IG-DMR are shown as colored circles. Small RNAs expressed by IG-DMR are indicated with blue lines. Numbers in parentheses indicate reference numbers.

The *Dlk1*-*Dio3* imprinted domain is an imprinted gene cluster on mouse chromosome 12 that consists of the protein-coding genes *Dlk1*, *Rtl1*, and *Dio3* expressed from the paternal allele and polycistronic non-coding genes *Gtl2*/*Meg3*, *Rtl1as*, *Rian*, and *Mirg*, which are expressed from the maternal allele (Fig 1A) [4–11]. Allele-specific expression of these genes is regulated by the *Dlk1*-*Meg3* intergenic DMR (IG-DMR), which is an ICR of the imprinted domain in which the paternal and maternal alleles are specifically methylated and unmethylated, respectively. However, because IG-DMR is a large 8.9 kb sequence, the full extent of the regulatory mechanisms of imprinted gene expression by IG-DMR remains largely unknown [12].

Mice lacking 4.1 kb of the IG-DMR die when the deletion allele (IG-DMR^KO^) is maternally inherited, suggesting that a sequence that is essential for the establishment and/or maintenance of the unmethylated state of the maternal allele is present within this 4.1 kb region [13]. In our previous report, a 216-bp tandem repeat sequence (IG-DMR-Rep, hereafter IG^Rep^) located on the 4.1 kb region of the IG-DMR was identified as a regulatory sequence because the paternal inheritance of the IG^Rep^ deletion (IG-DMR^ΔRep^) caused embryonic lethality due to an abnormal hypomethylation of the entire IG-DMR sequence [14]. IG^Rep^ contains multiple binding motifs for ZFP57, which is a zinc finger protein involved in the maintenance of DNA methylation imprints during preimplantation development [15], further supporting that IG^Rep^ is essential for the maintenance of DNA methylation imprints in paternal IG-DMR. Recently, IG^Rep^ was reported to be part of the CpG island (IG^CGI^) on the *Dlk1* side of IG-DMR (Fig 1B) [16]. However, the molecular functions of the other regions of IG^CGI^ are still unknown.

The positive region of histone H3K27ac is an active enhancer marker that was identified on the *Meg3* side of IG-DMR in embryonic stem cells. This region is the intergenic transcriptional regulatory element (IG^TRE^) for the distal enhancer activity of *Meg3* (Fig 1B). Aronson et al. showed that a lack of IG^TRE^ on maternal alleles results in biallelic expression of *Dlk1* and repression of *Meg3* in pluripotent cells. Similar changes were observed following artificial hypermethylation of IG^TRE^ in the maternal allele. These results indicated that the IG^TRE^ is important for the unmethylated state of the entire IG-DMR in the maternal allele [17]. Several molecular mechanisms involved in IG^TRE^ function have been reported. Kota et al. showed that RNA polymerase II binds to the internal sequence of IG^TRE^ and that multiple small RNAs are transcribed from this region. Knockdown of these small RNAs results in the hypermethylation of the maternal alleles of IG-DMR [18]. In addition, the transcription factors AFF3 and ZFP281 bind to IG^TRE^ and regulate the expression of *Meg3* [19,20]. These results suggest that IG^TRE^ may be responsible for embryonic lethality in the maternal alleles of IG-DMR^KO^. These effects were observed in pluripotent stem cells and were not clarified *in vivo*. Among these molecular mechanisms, the most important one for control by IG^TRE^ has not yet been identified.

When the IG-DMR^KO^ allele is paternally inherited, the methylation state of *Meg3*-DMR is established normally despite lacking IG^Rep^ and does not cause phenotypic abnormalities [13]. This strongly suggests that there are sequence(s) inside the 4.1 kb region deleted in the IG-DMR^KO^ allele that rescue the effect of the IG^Rep^ deletion on the paternal allele. Moreover, it is unknown whether the sequence that rescues the effects of the IG^Rep^ deletion is on IG^CGI^, IG^TRE^, or the small RNA-expressing region, and whether the AFF3/ZFP281 binding sequence is associated with this region.

In this study, we generated and analyzed mutant mice lacking IG^TRE^, the small RNA-expressing region, or the AFF3/ZFP281 binding sequence to examine the functions of IG^TRE^ in ontogeny and to determine whether these sequences are important for the epigenetic status of maternal alleles. In addition, we analyzed mice with simultaneous deletions of IG^TRE^ and IG^Rep^ to clarify their association with the imprinted status of the paternal alleles.

## Methods

### Preparation of sgRNA and Cas9

The sgRNA cloning vector was gifted by George Church (Addgene, Plasmid #41824). The sgRNAs were cloned into a sgRNA cloning vector as described previously [21]. The sgRNAs and Cas9 were transcribed *in vitro* using the mMESSAGE/mMACHINE T7 Transcription Kit (Ambion, Austin, TX, USA) and a PCR-amplified template containing T7 and sgRNA sequences. RNAs were purified using a MEGAclear Transcription Clean-Up Kit (Ambion). The primers listed in S1 Table in S1 File were used for cloning sgRNAs and template amplification in *in vitro* transcription reactions. The following recognition sequences of the sgRNAs were used in this study: sg1: 5´-ACACACGGTCCGTTACAGCCTGG-3′; sg2: 5′-GTCGATCGTGAACTGCAGCCTGG-3′; sg3: 5′-GGAGAATGCCTTGAGCACAGGGG-3′; sg4: 5′-AGGAGAAACCACTATAGCGTTGG-3′; and sg5: 5′-CTGTGGTCATCTTTACGGCCTGG-3′. The PAM sequences are underlined.

### Microinjection

For microinjection, fertilized eggs were collected from superovulated F_1_ hybrids of C57BL/6 and DBA/2 (BDF1) female mice crossed with BDF1 male mice. Embryos were incubated in KSOM medium (ARK resource, Kumamoto, Japan) at 37°C overnight. A mixture of two sgRNAs (100 ng/µL each) and Cas9 protein (100 ng/µL; Nippon Gene, Tokyo, Japan) was microinjected into the cytoplasm of the fertilized eggs. To generate mice carrying a serial deletion of IG^TRE^, a mixture of three sgRNAs (100 ng/µL) was microinjected together with Cas9 protein (100 ng/µL) into one blastomere of the two-cell embryos as previously described [22,23]. Injected two-cell embryos were transferred into pseudopregnant ICR female mice. Each deletion allele was isolated from the founder mice by backcrossing. All the mice were purchased from Sankyo Lab Service (Tokyo, Japan). All the animal experiments were approved by the Animal Care and Use Committee of the National Research Institute for Child Health and Development (Tokyo, Japan). All experiments were conducted following approved animal protocols. Mice were housed in a temperature and humidity-controlled environment with a 12 h light/dark cycle, all experiments were maintained under a 12-h light/dark cycle, and food and water were ad libitum. Mice were sacrificed by cervical dislocation. Mice were anesthetized with intraperitoneal injection of a mixture of 0.75 mg/kg body weight (b.w.) of medetomidine, 4.0 mg/kg b.w. midazolam and 5.0 mg/kg b.w butorphanol and its reversal by subcutaneous injection of 0.75 mg/kg b.w atipamezole.

### Genotyping analysis

To genotype the founder pups and embryos, genomic DNA was extracted from the fingertips or tail tips. PCR was performed using BIOTAQ (BioLine, London, UK) with the primers listed in S1 Table in S1 File. For sequencing, PCR products were treated with ExoSAP-IT (Affymetrix, Santa Clara, CA, USA) and sequenced on a capillary sequencer (3130xl and 3500xL Genetic Analyzers, Applied Biosystems, Foster City, CA, USA).

### Expression analysis

Expression analyses were performed as previously described [24]. Briefly, total RNA was isolated from embryos at 14.5 dpc using ISOGEN (Nippon Gene) and treated with TURBO DNase (Ambion). The cDNA was synthesized using Superscript II (Thermo Fisher Scientific, Waltham, MA USA) and an oligo dT primer. The reverse transcription-quantitative polymerase chain reaction (RT-qPCR) was performed using Power SYBR Green (Thermo Fisher Scientific). The relative quantification of imprinted gene expression was normalized to *Actb* expression levels and calculated using the ΔΔCt method.

To detect allelic expression of imprinted genes, IG-DMR^ΔRep+TRE/+^ embryos at 14.5 dpc were obtained by crossing IG-DMR^ΔRep+TRE^ female mice with JF1/Ms males. RT-PCR products were amplified using ExTaq HS (Takara Bio), treated with ExoSAP-IT, and sequenced. JF1/Ms mice were obtained from the National Institute of Genetics (NIG).

### DNA methylation analysis

Genomic DNA was extracted from the tail tips of the embryos at 14.5 dpc. Sodium bisulfite conversion of DNA was performed using the EpiTect Bisulfite Kit (QIAGEN). PCR was performed using primers targeting IG-DMR, *Meg3*-DMR, and EpiTaq HS (Takara Bio, Shiga, Japan). The amplified PCR products were cloned into a pGEM-T easy vector (Promega) and sequenced. Sequence data were analyzed using QUMA (http://quma.cdb.riken.jp/top/quma_main_j.html) [25]. For COBRA analysis, the PCR products were digested with TaqI (New England Biolabs, Ipswich, MA, USA). Black/white reversal of the gel image was performed using ImageJ (https://imagej.net/ij/index.html).

### Statistical analyses

All experiments were performed with at least two biological replicates. Statistical analyses were performed using an unpaired two-tailed *t*-test and a one-way ANOVA. *P*-values < 0.05 were considered significant.

## Results

### Deletion of IG^CGI^ disrupts imprinted gene expression on the paternal but not maternal allele

**Fig 2:**
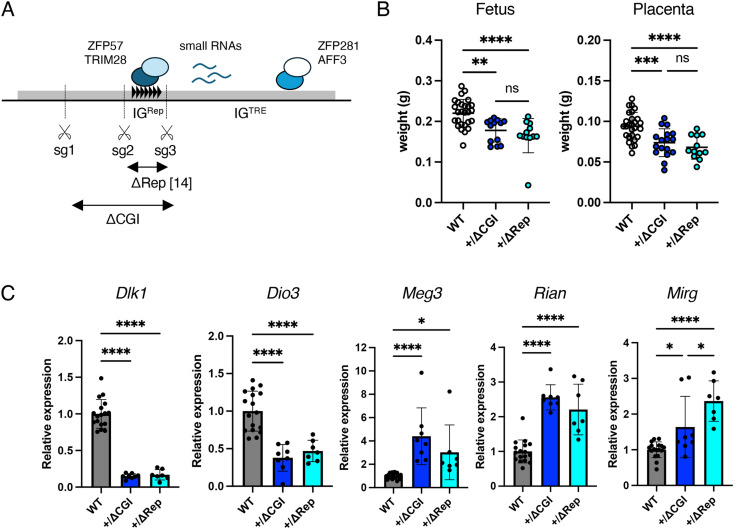
Phenotypes of IG-DMR^+/ΔCGI^ mice. (A) Schematic representation of IG-DMR. The gray box indicates IG-DMR. The repeated unit of IG^Rep^ is indicated by black triangles. Transcriptional factors that bind to IG-DMR are shown as colored circles. Small RNAs expressed by IG-DMR are indicated with blue lines. Scissors indicate the sg1-3 target sequences. (B) Fetal body and placental weights at 14.5 dpc. White, blue, and light blue dots indicate individual weights from WT (n = 27), IG-DMR^+/ΔCGI^ (n = 13), and IG-DMR^+/ΔRep^ (n = 12) mice, respectively. Black lines and error bars indicate the mean and standard deviation (SD), respectively. (C) Expression levels of imprinted genes. Gray, blue, and light blue bars indicate mean expression levels in WT (n = 27), IG-DMR^+/ΔCGI^ (n = 13), and IG-DMR^+/ΔRep^ (n = 12) embryos at 14.5 dpc, respectively. *: *P* < 0.05; **: *P* < 0.01; ***: *P* < 0.001; and ****: *P* < 0.0001 (unpaired two-tailed *t*-test). All samples were collected from 2–3 li*t*ters. Source data for Fig 2B are provided in Supplementary Information S2 Table in S1 File and S3 Table.

First, we created IG-DMR deletions in mice using the CRISPR/Cas9 system to identify the region responsible for the imprinting abnormalities and perinatal lethality in the maternal alleles observed in IG-DMR^KO^. Single-guide RNAs (sgRNAs) (sg1–4) were designed for the 4.1 kb region deleted in IG-DMR^KO^ mice [13]. The deletions were generated using sg1/sg3, which spans 1758 bp, including IG^Rep^ and IG^CGI^ (IG-DMR^ΔCGI^) (Fig 2A and S1 Fig in S1 File). Mice that inherited the deletion allele paternally (IG-DMR^+/ΔCGI^) and maternally (IG-DMR^ΔCGI/+^) were generated, and their phenotypes were analyzed. A ratio of the number of pups with IG-DMR^ΔCGI^ and wild-type (WT) alleles obtained from IG-DMR^ΔCGI/+^ female mice mated with WT male mice was close to the Mendelian ratio (Table 1). The gross phenotype of IG-DMR^ΔCGI/+^ mice was indistinguishable from WT and grew normally (S2 Fig in S1 File). However, the mating of IG-DMR^ΔCGI^ males with WT female mice produced no living pups (Table 1). To elucidate the effects of paternal transmission of the IG-DMR^ΔCGI^ allele in embryonic development, the phenotypes of IG-DMR^+/ΔCGI^ embryos were examined. IG-DMR^+/ΔCGI^ exhibited growth retardation at 14.5 dpc, which led to embryonic lethality at 18.5 dpc (Table 1). A reduction in placental weight was also observed from 14.5 dpc in IG-DMR^+/ΔCGI^ placenta when compared with WT. In addition, these phenotypes were comparable to those of IG-DMR^+/ΔRep^ (Fig 2B). Expression analysis of imprinted genes in IG-DMR^+/ΔCGI^ embryos at 14.5 dpc showed decreased expression of paternally expressed genes and increased expression of maternally expressed genes. Moreover, when the levels of imprinted gene expression were compared between IG-DMR^+/ΔCGI^ and previously reported IG-DMR^+/ΔRep^ embryos, the expression levels of almost all imprinted genes examined except *Mirg* were comparable between the two genetic mutant lines (Fig 2C). From these results, we concluded that the phenotype observed in IG-DMR^+/ΔCGI^ embryos is similar to that of IG-DMR^+/ΔRep^ and that maternal inheritance of IG^CGI^ deletions, including IG^Rep^, did not cause perinatal lethality.

**Table 1:** Mating test of ΔCGI and ΔRep + TRE mice.

Mutant	Transmission	no. of mutant (dead)/ total		
		14.5 dpc	18.5 dpc	newborn
ΔCGI	Maternal	2 (0)/ 9	NA	10 (0)/ 26
	Paternal	14 (4)/ 26	13 (5)/ 27	0 (0)/ 18
ΔRep + TRE	Maternal	10 (0)/ 27	NA	12 (0)/ 36
	Paternal	9 (0)/ 21	NA	24 (0)/ 40

### Maternal inheritance of a mutant allele simultaneously deleted IG^TRE^ and IG^Rep^ causing postnatal lethality

**Fig 3:**
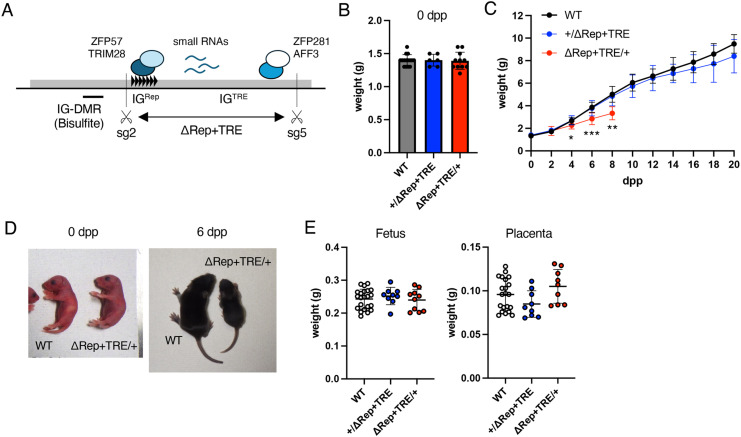
Phenotypes of IG-DMR^ΔRep^ ^+^^TRE^ **mice.** (A) Schematic representation of IG-DMR. The gray box indicates IG-DMR. The repeated unit of IG^Rep^ is indicated by black triangles. Transcriptional factors that bind to IG-DMR are shown as colored circles. Small RNAs expressed by IG-DMR are indicated with blue lines. Scissors indicate the target sequences of sg2 and sg5. PCR-amplified regions for bisulfite analysis of IG-DMR are indicated under the illustration. (B) Body weights of WT, IG-DMR^+/ΔRep+TRE^ and IG-DMR^ΔRep+TRE/+^ pups at 0 dpp (left, WT, IG-DMR^+/ΔRep+TRE^, and IG-DMR^ΔRep+TRE/+^; n = 15, n = 6, and n = 11, respectively). (C) Postnatal growth of WT, IG-DMR^+/ΔRep+TRE^, and IG-DMR^ΔRep+TRE/+^ pups. Sample numbers at each stage are shown in S2 Table in S1 File. Error bars indicate SD. *: *P* < 0.05; **: *P* < 0.01; ***: *P* < 0.001 (One-way ANOVA). (D) Representative photographs of WT and IG-DMR^ΔRep+TRE/+^ pups at 0 dpp (left) and 6 dpp (right). (E) Fetal body and placental weights at 14.5 dpc. White, blue, and red dots indicate individual weights of WT (n = 22), IG-DMR^+/ΔRep+TRE^ (n = 9), and IG-DMR^ΔRep+TRE/+^ (n = 10), respectively. Black lines and error bars indicate the mean and SD, respectively. All samples were collected from 2–3 litters. Source data for Fig 3B,C,E are provided in Supplementary Information S3 Table.

Next, mice lacking 3077 bp containing IG^Rep^ and IG^TRE^ (IG-DMR^ΔRep+TRE^) were generated using sg2/sg4 (Fig 3A and S1 Fig in S1 File). Mice that inherited the allele paternally (IG-DMR^+/ΔRep+TRE^) and maternally (IG-DMR^ΔRep+TRE/+^) were produced, and their phenotypes were analyzed. We observed that the ratio of the number of pups with IG-DMR^ΔRep+TRE^ and WT allele obtained from IG-DMR^ΔRep+TRE^ males crossed with WT female mice was close to the Mendelian ratio, while the gross phenotypes of IG-DMR^+/ΔRep+TRE^ mice were indistinguishable from WT and grew normally, suggesting that simultaneous deletion of IG^TRE^ and IG^Rep^ rescued the lethal phenotype caused by IG^Rep^ deletion on the paternal allele (Fig 3B). Conversely, IG-DMR^ΔRep+TRE/+^ pups were born normally but exhibited gradual growth retardation from 4 d postpartum (dpp), and all pups died within 14 dpp (Fig 3B-D). Moreover, fetal and placental weights of IG-DMR^+/ΔRep+TRE^ and IG-DMR^ΔRep+TRE/+^ at 14.5 dpc were not significantly different from those of the WT littermates (Fig 3E).

**Fig 4:**
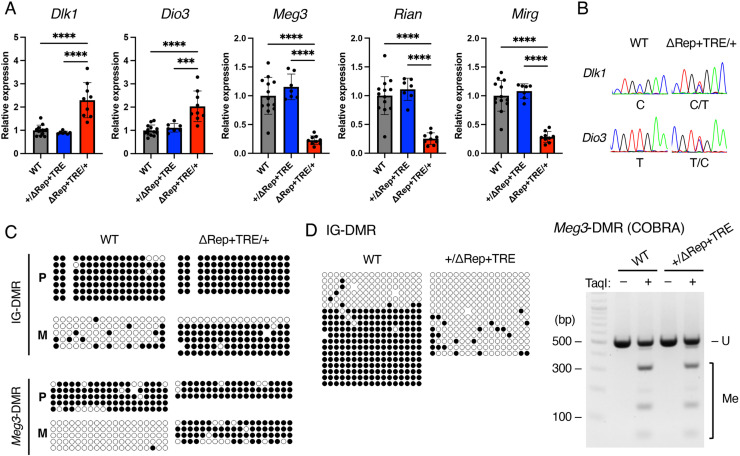
Expression of imprinted genes and DNA methylation in IG-DMR^ΔRep^ ^+^^TRE^ **mice.** (A) Expression levels of imprinted genes. Gray, blue, and red bars indicate mean expression levels in WT (n = 13), IG-DMR^+/ΔRep+TRE^ (n = 7), and IG-DMR^ΔRep+TRE/+^ (n = 9) embryos at 14.5 dpc, respectively. ***: *P* < 0.001; ****: *P* < 0.0001 (unpaired two-tailed *t*-tes*t*). All samples were collected from 2–3 litters. (B) Representative electropherograms of *Dlk1* and *Dio3* amplified from WT and IG-DMR^ΔRep+TRE/+^ cDNAs are shown. Polymorphic nucleotides are shown below the electropherograms. (C) DNA methylation analysis of IG-DMR and *Meg3*-DMR in WT and IG-DMR^ΔRep+TRE/+^ mice. Representative results of the bisulfite analysis are shown. Black and white circles indicate methylated and unmethylated CpG sites, respectively. “P” and “M” indicate paternal and maternal alleles, respectively. (D) DNA methylation analysis of IG-DMR and *Meg3*-DMR in WT and IG-DMR^+/ΔRep+TRE^. (left) Representative results of the bisulfite analysis of IG-DMR. (right) Representative gel of co-bisulfite restriction analysis (COBRA) at *Meg3*-DMR in WT and IG-DMR^+/ΔRep+TRE^. Source data for Fig 4A are provided in Supplementary Information S3 Table.

To ask if the postnatal lethality observed in IG-DMR^ΔRep+TRE/+^ is caused by aberrant expression of imprinted genes, the expression levels of imprinted genes were analyzed in IG-DMR^ΔRep+TRE/+^ embryos at 14.5 dpc. We observed that the expression of maternally expressed genes decreased and that of paternally expressed genes increased in IG-DMR^ΔRep+TRE/+^ embryos, whereas no significant changes were observed in IG-DMR^+/ΔRep+TRE^ embryos (Fig 4A). To examine whether increased expression of *Dlk1* and *Dio3* in IG-DMR^ΔRep+TRE/+^ is a result of biallelic expression, allelic expression was analyzed in embryos obtained from mating between IG-DMR^+/ΔRep+TRE^ females and JF1/Ms males. As expected, sequencing analysis showed that biallelic expression of paternally expressed genes was observed in IG-DMR^ΔRep+TRE/+^ embryos (Fig 4B), further indicating that the postnatal lethality observed in IG-DMR^ΔRep+TRE/+^ was due to aberrant expression of imprinted genes. Moreover, bisulfite sequencing analysis of IG-DMR and *Meg3*-DMR in IG-DMR^ΔRep+TRE/+^ embryos showed that both IG-DMR and *Meg3*-DMR on the paternal allele were highly methylated (Fig 4C), indicating that the sequence responsible for perinatal lethality in the maternal allele is IG^TRE^.

### Deletion of IG^TRE^ rescues the phenotype of IG^Rep^ deletion on the paternal allele

Since no phenotype was observed in IG-DMR^+/ΔRep+TRE^ mice despite the lack of IG^Rep^ on the paternal allele, we hypothesized that the simultaneous deletion of IG^TRE^ and IG^Rep^ would rescue the phenotypes of IG-DMR^+/ΔRep^ mice. We assessed the imprinted genes’ expression in IG-DMR^+/ΔRep+TRE^ embryos at 14.5 dpc; RT-qPCR analysis showed that the expression levels of all imprinted genes in IG-DMR^+/ΔRep+TRE^ embryos were comparable to those in the WT littermates (Fig 4A). DNA methylation analysis showed that IG-DMR was hypomethylated, while methylation of *Meg3*-DMR was restored to a level similar to that of the WT, indicating that even though IG^Rep^ was deleted and the IG-DMR on the paternal allele was hypomethylated, the simultaneous deletion of IG^TRE^ and IG^Rep^ resulted in a normal methylation level of *Meg3*-DMR (Fig 4D and S4 Fig in S1 File). These results suggested that the simultaneous deletion of IG^TRE^ and IG^Rep^ establishes DNA methylation imprints in *Meg3*-DMR, even though IG-DMR is hypomethylated.

### Creation and phenotypic analysis of IG^TRE^ deletion mice

**Fig 5:**
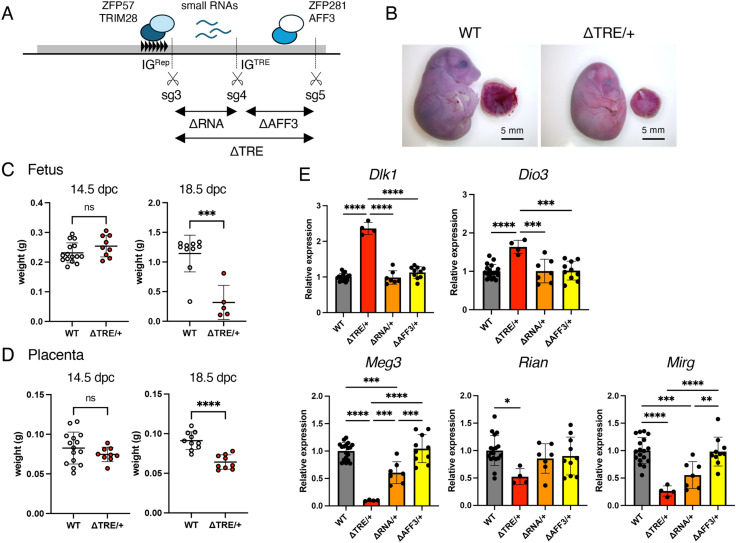
Phenotypes of IG-DMR^ΔTRE/^ ^+^**, IG-DMR**^ΔRNA/+^**, and IG-DMR**^ΔAFF3/+^ **mice.** (A) Schematic representation of IG-DMR. The gray box indicates IG-DMR. The repeated unit of IG^Rep^ is indicated by black triangles. Transcriptional factors that bind to IG-DMR are shown as colored circles. Small RNAs expressed by IG-DMR are indicated with blue lines. Scissors indicate the target sequences of sg3-5. (B) Representative photographs of IG-DMR^ΔTRE/+^ and WT littermate embryos at 18.5 dpc. Scale bar: 5 mm. (C) Fetal body weights of WT and IG-DMR^ΔTRE/+^ embryos at 14.5 (left, WT and IG-DMR^ΔTRE/+^; n = 15 and n = 9, respectively) and 18.5 dpc (right, WT and IG-DMR^ΔTRE/+^; n = 10 and n = 5, respectively). (D) Placental weights of WT and IG-DMR^ΔTRE/+^ at 14.5 (left, WT and IG-DMR^ΔTRE/+^; n = 15 and n = 9, respectively) and 18.5 dpc (right, WT and IG-DMR^ΔTRE/+^; n = 10 and n = 5, respectively). White and red dots indicate individual weights of WT and IG-DMR^ΔTRE/+^, respectively. The mean and SD are shown as black lines and error bars, respectively. ***: *P* < 0.001; ****: *P* < 0.0001 (unpaired two-tailed t-test). ns: not significant. All samples were collected from two litters. (E) Expression of imprinted genes in IG-DMR^ΔTRE/+^, IG-DMR^ΔRNA/+^, and IG-DMR^ΔAFF3/+^ mice. Gray, red, orange, and yellow bars indicate mean expression levels in WT (n = 18), IG-DMR^ΔTRE/+^ (n = 4), IG-DMR^ΔRNA/+^ (n = 7), and IG-DMR^ΔAFF3/+^ (n = 10) embryos at 14.5 dpc, respectively. Error bars indicate SD. *: *P* < 0.05; **: *P* < 0.01; ***: *P* < 0.001; ****: *P* < 0.0001 (One-way ANOVA). All samples were collected from two litters. Source data for Fig 5C-E are provided in Supplementary Information S3 Table.

We designed another sgRNA (sg5) between sg3 and sg4 to elucidate the roles of IG^TRE^, a small RNA-expressing region, and the AFF3/ZFP281 binding sequence. Cas9 and sg3, 4, and 5 were mixed together and microinjected into fertilized eggs to create mice lacking 1578 bp that contained the small RNA-expressing region (IG-DMR^ΔRNA^), 1070 bp of the binding sequence for AFF3 and ZFP281 (IG-DMR^ΔAFF3^), and 2631 bp of the entire IG-DMR (IG-DMR^ΔTRE^) (Fig 5A and S3 Fig in S1 File). Phenotypic analyses were performed in mice that inherited these alleles paternally or maternally, which showed that when male mice with the IG-DMR^ΔTRE^ allele were crossed with WT female mice, a ratio of the number of pups with the deletion or the WT allele was close to the Mendelian ratio. However, when female mice with the IG-DMR^ΔTRE^ allele were mated with WT male mice, all individuals obtained were WT, and no mice with the deletion (IG-DMR^ΔTRE/+^) were obtained (Table 2). To test whether maternal transmission of the IG-DMR^ΔTRE^ allele caused embryonic lethality, we examined the phenotypes of IG-DMR^ΔTRE/+^ mice during embryonic development. Although fetal and placental weights in IG-DMR^ΔTRE/+^ embryos at 14.5 dpc were not significantly different from those of WT littermates, severe growth retardation was observed in IG-DMR^ΔTRE/+^ at 18.5 dpc (Fig 5B-D).

**Table 2:** Mating test of ΔRNA, ΔAFF3, and ΔTRE mice.

Mutant	Transmission	no. of mutant (dead)/ total		
		14.5 dpc	18.5 dpc	newborn
ΔRNA	Maternal	9 (0)/ 26	NA	21 (0)/ 39
	Paternal	NA	NA	5 (0)/ 10
ΔAFF3	Maternal	10 (0)/ 15	NA	13 (0)/ 26
	Paternal	NA	NA	5 (0)/ 10
ΔTRE	Maternal	9 (0)/ 24	8 (6)/ 19	0 (0)/ 16
	Paternal	NA	NA	12 (0)/ 29

Next, we examined the phenotypes of pups with maternally inherited IG-DMR^ΔRNA^ and IG-DMR^ΔAFF3^ alleles (IG-DMR^ΔRNA/+^ and IG-DMR^ΔAFF3/+^). When female mice with the IG-DMR^ΔRNA^ or IG-DMR^ΔAFF3^ allele were crossed with WT males, pups were obtained according to the Mendelian ratio, and no gross phenotypes were observed in these pups (Table 2). The same results were obtained when male mice with the IG-DMR^ΔRNA^ or IG-DMR^ΔAFF3^ allele were crossed with WT females (Table 2). Similarly, IG-DMR^ΔRNA/+^ and IG-DMR^ΔAFF3/+^ embryos did not show growth retardation or embryonic lethality (S4 Fig in S1 File).

To clarify the effects of the IG^TRE^ deletions and its internal sequences on imprinted gene expression, 14.5 dpc embryos from IG-DMR^ΔTRE/+^, IG-DMR^ΔRNA/+^, and IG-DMR^ΔAFF3/+^ mice were generated. RT-qPCR showed that paternally expressed genes were overexpressed while maternally expressed genes were downregulated in IG-DMR^ΔTRE/+^ when compared to that of WT. In the IG-DMR^ΔRNA/+^ embryos, the expression levels of paternally expressed genes *Dlk1* and *Dio3* were not significantly different from those of WT, while the expression levels of maternally expressed genes *Meg3* and *Mirg* were downregulated to approximately half of those of WT. In IG-DMR^ΔAFF3/+^ embryos, *Rtl1* was upregulated compared to that of WT, but the expression levels of other paternally expressed genes were not significantly different from those of WT. Similarly, the expression of *Meg3*, *Rian*, and *Mirg* was not significantly different in IG-DMR^ΔAFF3/+^ embryos when compared to that of WT (Fig 5E).

Our results indicated that the entire IG^TRE^ region is essential for the regulation of imprinted gene expression in maternal alleles. Moreover, the region where small RNAs are transcribed and the region that contains the AFF3/ZFP281-binding motif are functionally redundant in the regulation of maternally expressed genes during embryogenesis.

## Discussion

In this study, we showed that IG^TRE^ deletion causes embryonic lethality through the abnormal expression of parentally imprinted genes. These results suggest that the loss of IG^TRE^ is the cause of lethality in maternally inherited IG-DMR^KO^. Moreover, we showed that deletion of the small RNA-expressing region and AFF3/ZFP281 binding sequence in the IG^TRE^ alone does not cause an obvious phenotype in deletion mouse lines, suggesting these regions are functionally redundant for IG^TRE^ function.

IG^TRE^ is a putative distal enhancer that regulates the maternal-specific expression of lncRNAs in pluripotent cells [18–20,26]. Consistent with previous reports, our data showed that IG^TRE^ is essential for the expression of maternally expressed genes *in vivo*. Moreover, we observed that deletion of small RNA-expressing region in the maternal allele resulted in decreased expression of *Meg3* and *Mirg*. The molecular mechanisms of gene expression *in vivo* are unknown, but an association with pluripotency transcription factors such as OCT4, SOX2, and KLF4 is possible [26], as these factors bind to IG^TRE^ and are implicated in the association between the unmethylated state of the maternal allele and enhancer activity in the region with a complex that forms between these factors in pluripotent cells [26,27]. In addition, the *Igf2*-*H19* imprinted locus was shown to bind the pluripotency transcription factors SOX2 and OCT4 to its ICR, with H19-ICR important for the unmethylated state of the maternal allele [28,29]. These results suggest that the binding of pluripotent factors to the small RNA-expressing region in IG^TRE^ may be essential for maintaining the unmethylated state of IG-DMR and enhancer activity. Since the molecular mechanisms of pluripotent transcription factors for the unmethylated state of ICRs are still unclear, further studies are required to elucidate the molecular mechanisms by which this region regulates DNA methylation status and the transcription of maternally expressed genes.

The deletion of the small RNA-expressing region and the AFF3/ZFP281 binding sequence in IG^TRE^ alone did not cause an obvious phenotype in our deletion mice lines, suggesting that IG^TRE^ causes the lethality in maternally inherited IG-DMR^KO^ and that the small RNA-expressing region and the AFF3/ZFP281-binding sequence are functionally redundant for IG^TRE^ function *in vivo*. In contrast, AFF3/ZFP281 binding to the IG-DMR regulates the polycistronic expression of maternally expressed genes in embryonic stem (ES) cells [19,20]. However, our results showed that deletion of the AFF3/ZFP281 binding site did not affect the expression of maternally expressed genes *in vivo*. These results suggested that the effect of AFF3/ZFP281 binding on the expression of maternally expressed genes is limited in a temporal and/or cell type-specific manner.

**Fig 6:**
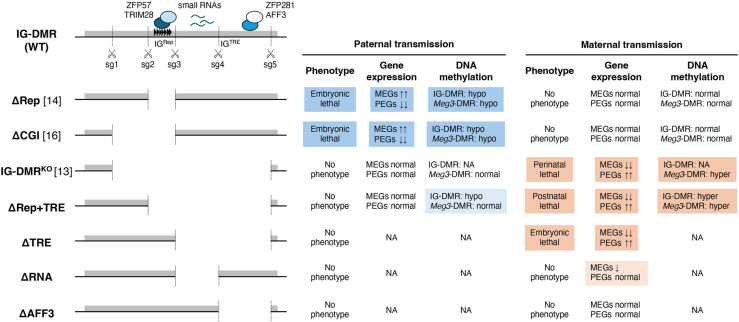
Summary of this study. Summary of the results from the mutant mice. Deleted regions in IG-DMR that each mutant mouse harbors are shown on the left side. The numbers in parentheses indicate the reference numbers. Panels showing significant differences are highlighted. The arrows indicate increased or decreased. PEGs: paternally expressed genes; MEGs: maternally expressed genes; hypo: hypomethylated; hyper: hypermethylated; NA: not analyzed.

The 4.1 kb region of the IG-DMR has two features: 1) it is essential for the unmethylated state of the maternal allele, and 2) it can rescue the loss of IG^Rep^ on the paternal allele [13]. The deletion of IG^CGI^ in the paternal allele causes embryonic lethality but does not show a gross phenotype when the deletion allele is maternally inherited [16]. These findings are consistent with those obtained from the IG-DMR^+/ΔCGI^ mice we generated. In addition, we showed that the expression levels of the imprinted genes in IG-DMR^+/ΔCGI^ mice were similar to those of IG-DMR^+/ΔRep^ mice. This result suggests that the region between sg1 and sg3 in IG^CGI^ is not required for both the paternal and maternal alleles. However, IG-DMR^ΔRep+TRE/+^ mice had aberrant expression of imprinted genes and hypermethylation at IG-DMR on the maternal allele, while the IG-DMR^+/ΔRep+TRE^ mice did not exhibit any phenotypic defects or aberrant imprinted gene expression. These results indicated that the region between sg2 and sg4 containing IG^Rep^ and IG^TRE^ is responsible for recapitulating the two features of the 4.1 kb region (Fig 6).

Our results revealed that the deletion of IG^TRE^ rescued the embryonic lethality caused by the paternally inherited deletion of IG^Rep^. Although it has been believed that the methylation status of the *Meg3*-DMR on the paternal allele is established according to the methylation status of the IG-DMR, the *Meg3*-DMR was normally methylated even though the IG-DMR in the paternal allele was hypomethylated in IG-DMR^+/ΔRep+TRE^ embryos. Previously, we and others have reported that both IG^TRE^ and *Meg3*-DMR are hypomethylated in mice lacking IG^Rep^ in the paternal allele [14,22], and that paternal *Meg3*-DMR is normally methylated in IG-DMR^+/KO^ mice [13]. These results suggest that *Meg3*-DMR could be methylated in the absence of the unmethylated IG^TRE^ on the paternal allele, which is consistent with a previous report on epigenome editing in ES cells [30]. In contrast, the presence of unmethylated IG^TRE^ on maternal alleles is important for maintaining the unmethylated states of IG^Rep^ and *Meg3*-DMR. However, it is unclear how the enhancer activity of IG^TRE^ is associated with the unmethylated status of the maternal allele and requires further studies.

In summary, our findings reveal that IG^TRE^ is responsible for the lethal phenotype of the maternally inherited 4.1 kb deletion of IG-DMR. Because deletions or epimutations of the IG-DMR on the maternal allele can cause Kagami-Ogata syndrome in humans [31], our findings contribute to a better understanding of the pathogenesis of this disease.

## Supporting information

S1 File **S1 Fig.** Nucleotide sequences of mutated loci in IG-DMR^ΔCGI^ and IG-DMR^ΔRep+TRE^ mice. **S2 Fig.** Postnatal growth of IG-DMR^ΔCGI/+^ mice and their WT littermates. **S3 Fig.** Nucleotide sequences of mutated loci in IG-DMR^ΔTRE^, IG-DMR^ΔRNA^, and IG-DMR^ΔAFF3^ mice.**S4 Fig.** Phenotypes of IG-DMR^ΔRNA/+^ and IG-DMR^ΔAFF3/+^ mice. **S5 Fig**. A full gel image of COBRA analysis is provided in Fig 4D. **S1 Table.** Primer information. **S2 Table.** Sample sizes of each stage in the growth curve.
(PDF)
