# Supplementary material for: Identification of maternal allele sequences of IG-DMR that are essential for neonatal viability
Source: PLoS One. 2025 May 22;20(5):e0324882. doi: 10.1371/journal.pone.0324882 (PMC12097578; doi:10.1371/journal.pone.0324882)
Supplement: S1 File — S1 Fig. Nucleotide sequences of mutated loci in IG-DMRΔCGI and IG-DMRΔRep+TRE mice. S2 Fig. Postnatal growth of IG-DMRΔCGI/+ mice and their WT littermates. S3 Fig. Nucleotide sequences of mutated loci in IG-DMRΔTRE, IG-DMRΔRNA, and IG-DMRΔAFF3 mice.S4 Fig. Phenotypes of IG-DMRΔRNA/+ and IG-DMRΔAFF3/+ mice. S5 Fig. A full gel image of COBRA analysis is provided in Fig 4D. S1 Table. Primer information. S2 Table. Sample sizes of each stage in the growth curve. (PDF) [file pone.0324882.s001.pdf]

**Supplemental information**

# Identification of maternal allele sequences of IG-DMR that are essential for neonatal viability

Satoshi Hara[1,2], Akari Muramatsu[1,3], Miho Terao[1], and Shuji Takada[1,3,*]

[1]Department of Systems BioMedicine, National Research Institute for Child Health and Development, Tokyo 157-8535, Japan
[2]Devision of Molecular Genetics & Epigenetics, Department of Biomolecular Science, Faculty of Medicine, Saga University, Saga 849-8501, Japan
3Department of NCCHD, Graduate School of Medical and Dental Sciences, Institute of Science Tokyo, Tokyo 113 – 8510, Japan

[*]Correspondence should be addressed to Shuji Takada (takada-s@ncchd.go.jp, 2-10-1 Okura, Setagaya-ku, Tokyo 157-8535, Japan, Tel & Fax: +81-3-3417-2498).

# S1 Figure

IG-DMR$^{\Delta CGI}$

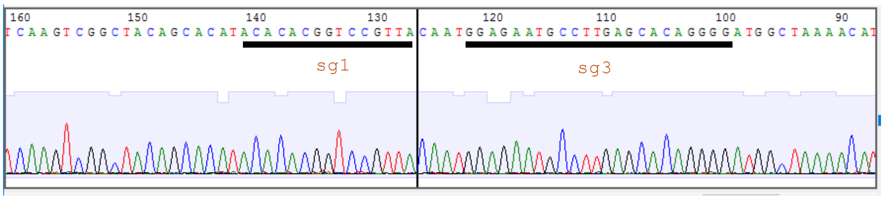


```
      sg1                                                                                                                        sg3
ACACACGGTCCGTTACAGCCTGGTACACCGGATGTGCAGCAGCAAAGCTAAGTGTGACAC…TCCTGGAGTGAGGGAAGGGCTGCATTATTTTGTCAATGGAGAATGCCTTGAGCACAGGGGATG
ACACACGGTCCGTTA---------------------------------------------…------------------------------------CAATGGAGAATGCCTTGAGCACAGGGGATG
```

1758 bp del

IG-DMR$^{\Delta Rep+TRE}$

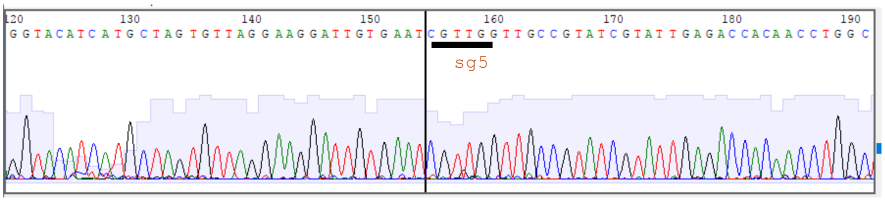


```
                                                  sg2                                          sg5
AGGATTGTGAATCTATACGGAGATGTGCTGTGGACCCAGGCTGCAGTTCACGATCGACTAG…CTAGCCCAGGAGAAACCACTATAGCGTTGGTTGCCGTATCGTATTGAGACCACAACCTGGCT
AGGATTGTGAAT-------------------------------------------------…------------------------CGTTGGTTGCCGTATCGTATTGAGACCACAACCTGGCT
```

3077 bp del

**S1 Figure. Nucleotide sequences of mutated loci in IG-DMR$^{\Delta CGI}$ and IG-DMR$^{\Delta Rep+TRE}$ mice.** Electropherograms are shown in the top panel. A part of the sgRNA sequence is underlined. A comparison of nucleotide sequences between WT and a mutant is shown in the bottom panel. The size of the deleted sequence is indicated with “del.”

**S2 Figure**

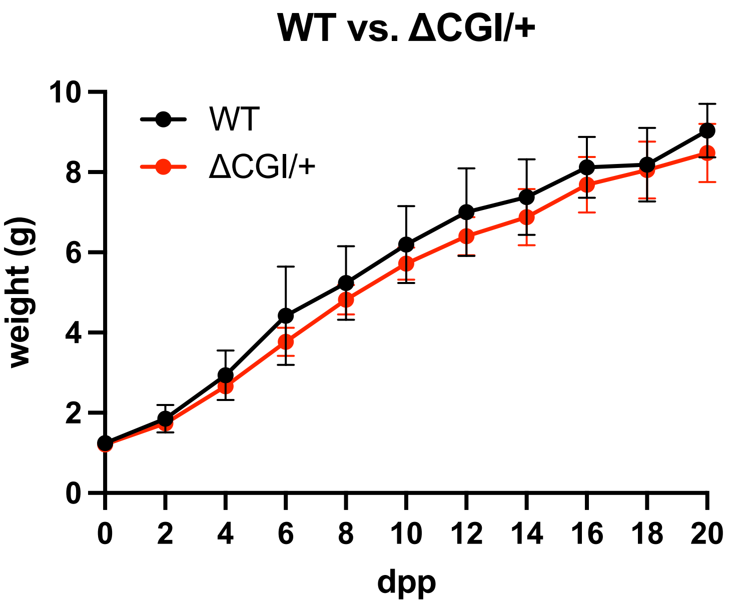


**S2 Figure. Postnatal growth of IG-DMR$^{\Delta CGI/+}$ mice and their WT littermates.** Source data for S2 Figure is in Supplementary information S2 Table.

# S3 Figure

IG-DMR$^{\Delta TRE}$

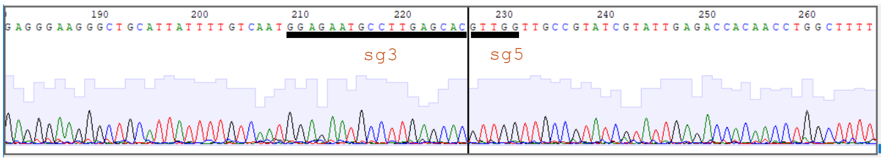


sg3 sg5

TTTTGTCAATGGAGAATGCCTTGAGCACAGGGGATGGCTAAAACATTCTCA…GCCCAGGAGAAACCACTATAGCGTTGGTTGCCGTATCGTATTGAGA

TTTTGTCAATGGAGAATGCCTTGAGCAC----------------------…----------------------GTTGGTTGCCGTATCGTATTGAGA

2631 bp del

IG-DMR$^{\Delta RNA}$

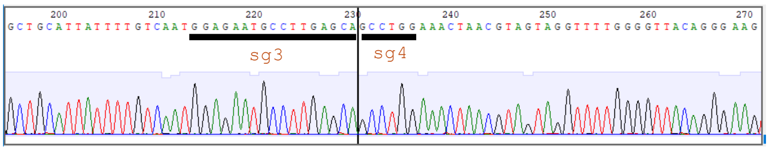


sg3 sg4

TTATTTTGTCAATGGAGAATGCCTTGAGCACAGGGGATGGCTAAAA…GACTTCCTGTGGTCATCTTTACGGCCTGGAAACTAACGTAGTAGGTTTTGGGG

TTATTTTGTCAATGGAGAATGCCTTGAGCA----------------…----------------------GCCTGGAAACTAACGTAGTAGGTTTTGGGG

1578 bp del

IG-DMR$^{\Delta AFF3}$

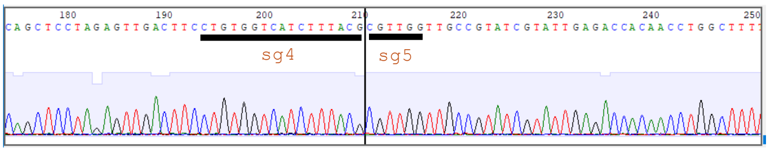


sg4 sg5

CTACAGCTCCTAGAGTTGACTTCCTGTGGTCATCTTTACGGCCTGGAAACTAACGTA…GAGAAACCACTATAGCGTTGGTTGCCGTATCGTATTGAGA

CTACAGCTCCTAGAGTTGACTTCCTGTGGTCATCTTTACG-----------------…---------------CGTTGGTTGCCGTATCGTATTGAGA

1070 bp del

**S3 Figure. Nucleotide sequences of mutated loci in IG-DMR$^{\Delta TRE}$, IG-DMR$^{\Delta RNA}$, and IG-DMR$^{\Delta AFF3}$ mice.** Electropherograms are shown at the top of the panel. A part of the sgRNA sequence is underlined. A comparison of nucleotide sequences between WT and a mutant is shown at the bottom of the panel. The size of the deleted sequence is indicated with "del."

**S4 Figure**

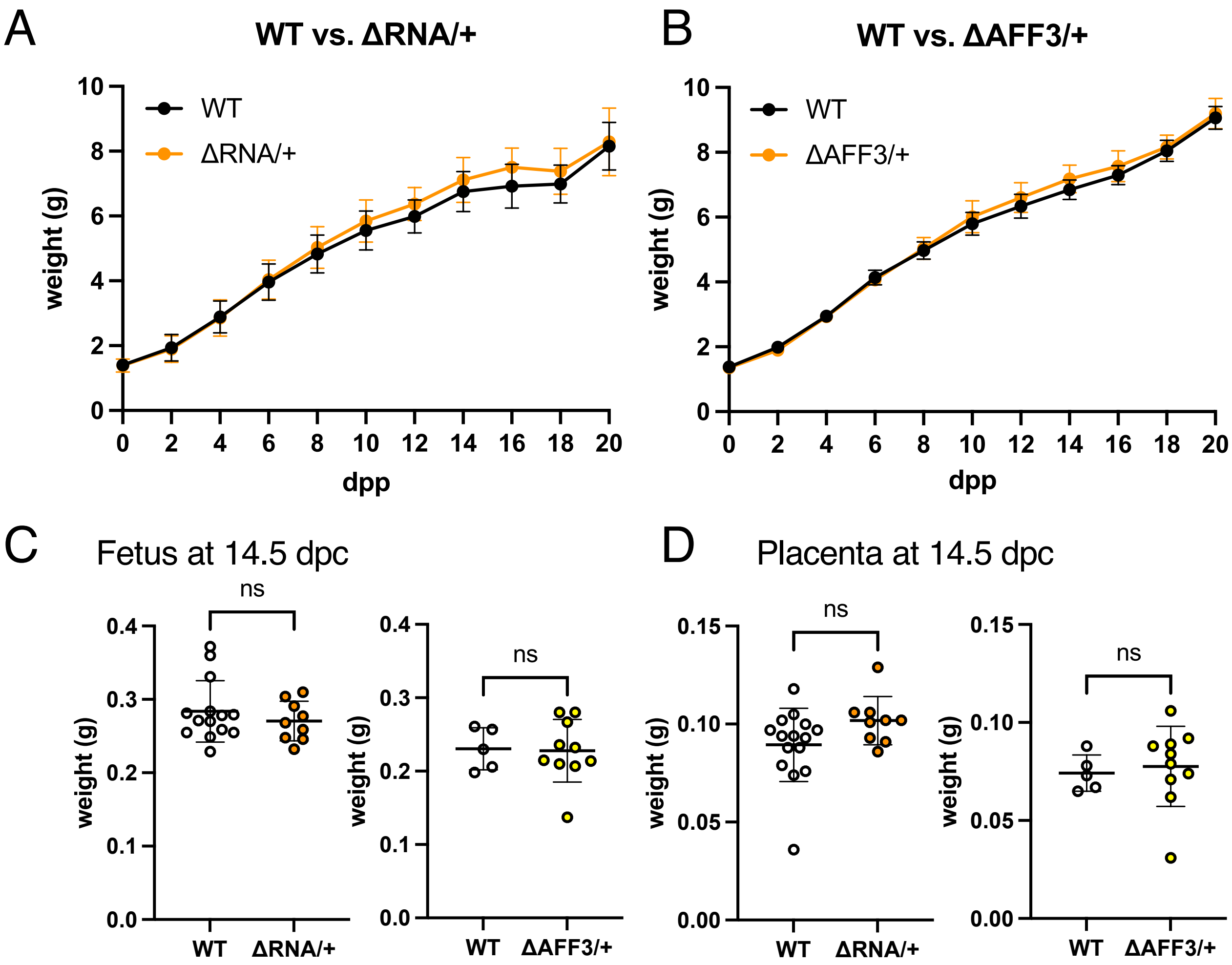


**S4 Figure. Phenotypes of IG-DMR$^{\Delta RNA/+}$ and IG-DMR$^{\Delta AFF3/+}$ mice.** (A) (left) Postnatal growth of IG-DMR$^{\Delta RNA/+}$ and WT littermates. (B) Postnatal growth of IG-DMR$^{\Delta AFF3/+}$ and WT littermates. (C) (left) Fetal body weights of IG-DMR$^{\Delta RNA/+}$ (n = 9) and WT (n = 14) littermate embryos at 14.5 dpc. (right) Fetal body weights of IG-DMR$^{\Delta AFF3/+}$ (n = 10) and WT (n = 5) littermate embryos at 14.5 dpc. (D) (left) Placental weights of IG-DMR$^{\Delta RNA/+}$ (n = 9) and WT (n = 14) littermates at 14.5 dpc. (right) Placental weights of IG-DMR$^{\Delta AFF3/+}$ (n = 10) and WT (n = 5) littermates at 14.5 dpc. Source data for S4 Figure is in Supplementary information S2 Table and S3 Table.

## S5 Figure

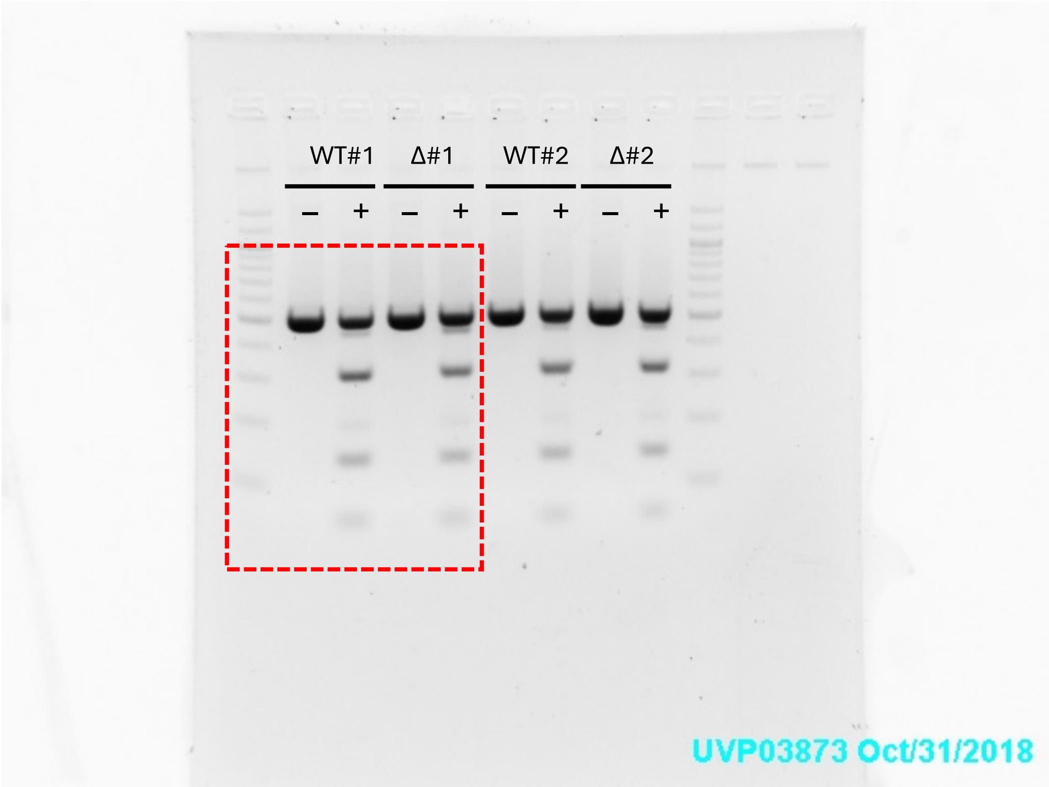


**S5 Figure. A full gel image of COBRA analysis is provided in Figure 4D.** The cropped region is indicated by a red-dotted box.

## S1 Table

| S1 Table. Primer information | |
|---|---|
| Primer name | Sequences (5'-3') |
| Primers for sgRNA preparation | |
| IG-DMRgRNA-A_F | CACACGGTCCGTTACAGCCgttttagagctagaaatagcaag |
| IG-DMRgRNA-A_R | aacGGCTGTAACGGACCGTGTGcggtgtttcgtcctttccac |
| IG-DMRgRNA-A_IVT_F | ttaatacgactcactataggCACACGGTCCGTTACAGCC |
| IG-DMRgRNA-B_F | GGAGAAACCACTATAGCGTgttttagagctagaaatagcaag |
| IG-DMRgRNA-B_R | aacACGCTATAGTGGTTTCTCCcggtgtttcgtcctttccac |
| IG-DMRgRNA-B_IVT_F | ttaatacgactcactataggGGAGAAACCACTATAGCGT |
| IG-DMRgRNA-M_F | GGCTGCAGTTCACGATCGAGTTTTAGAGCTAGAAATAGCAAG |
| IG-DMRgRNA-M_R | AACTCGATCGTGAACTGCAGCCCGGTGTTTCGTCCTTTCCAC |
| IG-DMRgRNA-M_IVT_F | TTAATACGACTCACTATAGGGGCTGCAGTTCACGATCGA |
| IG-DMRgRNA-N_F | GAGAATGCCTTGAGCACAGGTTTTAGAGCTAGAAATAGCAAG |
| IG-DMRgRNA-N_R | AACCTGTGCTCAAGGCATTCTCCGGTGTTTCGTCCTTTCCAC |
| IG-DMRgRNA-N_IVT_F | TTAATACGACTCACTATAGGGAGAATGCCTTGAGCACAG |
| IG-DMRgRNA-O_F | TGTGGTCATCTTTACGGCCgttttagagctagaaatagcaag |
| IG-DMRgRNA-O_R | aacGGCCGTAAAGATGACCACAcggtgtttcgtcctttccac |
| T7_IG-DMRgRNA-O | ttaatacgactcactataggTGTGGTCATCTTTACGGCC |
| Primers for genotyping | |
| ΔTR_geno_F | GAGTCCTATCATCCTGTATG |
| ΔTR_geno_R | GTCTGTATGGTCACAGCACC |
| AN_geno_F | AAGTAACAGGCTCTCACTGG |
| AN_geno_R | CAAGTACCAGATTCCATCA |
| nested_ geno_BF2 | GGCATATCCCATACCAAGCA |
| nested_ geno_BR2 | CAAGAGAGAAGGCAGGGTGT |
| Nested_geno_NF1 | CTATGGACTGGTGCCAAGGT |
| Nested_geno_OR1 | TGACAGGCTGCAGTAAATGG |
| Nested_geno_OF2 | ACAATTCCCACTTGATTTACGG |
| AM_geno_R1 | AGAAACAGCATAGCATAGCG |
| Nested geno PF1 | TAACTAAGCTGCCCCCAAAA |
| Nested geno QR1 | GCTGAGGCTTCCTTGTTCTG |

## S2 Table

| S2 Table. Sample sizes of each stage in the growth curve | | | | | | | | | | | |
|---|---|---|---|---|---|---|---|---|---|---|---|
| Figure 2B | | | | | | | | | | | |
| dpp | 0 | 2 | 4 | 6 | 8 | 10 | 12 | 14 | 16 | 18 | 20 |
| WT n | 15 | 15 | 15 | 15 | 15 | 15 | 15 | 14 | 15 | 13 | 10 |
| ΔCGI/+ n | 10 | 10 | 10 | 10 | 10 | 10 | 10 | 10 | 10 | 10 | 8 |
| | | | | | | | | | | | |
| S2 Figure | | | | | | | | | | | |
| dpp | 0 | 2 | 4 | 6 | 8 | 10 | 12 | 14 | 16 | 18 | 20 |
| WT n | 16 | 16 | 16 | 16 | 16 | 16 | 16 | 16 | 16 | 9 | 16 |
| ΔRep+TRE/+ n | 6 | 6 | 6 | 6 | 6 | 6 | 6 | 6 | 6 | 4 | 6 |
| +/ΔRep+TRE n | 11 | 3 | 4 | 5 | 3 | | | | | | |
| | | | | | | | | | | | |
| S4 Figure A | | | | | | | | | | | |
| dpp | 0 | 2 | 4 | 6 | 8 | 10 | 12 | 14 | 16 | 18 | 20 |
| WT n | 18 | 18 | 18 | 18 | 18 | 18 | 14 | 18 | 18 | 10 | 10 |
| ΔRNA/+ n | 20 | 20 | 20 | 19 | 19 | 19 | 15 | 19 | 19 | 10 | 10 |
| | | | | | | | | | | | |
| S4 Figure B | | | | | | | | | | | |
| dpp | 0 | 2 | 4 | 6 | 8 | 10 | 12 | 14 | 16 | 18 | 20 |
| WT n | 13 | 13 | 13 | 13 | 13 | 13 | 13 | 13 | 13 | 13 | 13 |
| ΔAFF3/+ n | 13 | 13 | 13 | 13 | 13 | 13 | 13 | 13 | 13 | 13 | 13 |
